# Digitalisation of staff health record system in MT Alvernia Hospital

**DOI:** 10.1017/ash.2025.77

**Published:** 2025-09-03

**Authors:** Calis Lim

## Abstract

**Background/Aim:** The manual compilation of employees’ health information and record-keeping can be an arduous and distressing task. Therefore, it is essential to invest time and effort in finding a solution to collect, store, and retrieve data in real-time when required. **Method:** To address this challenge, the Infection Control Team collaborated with the IT department and relevant HODs to develop an integrated and digitalized record system known as the “Staff Health System.” This system can be easily accessed by all employees, supervisors, and the infection control team. The Infection Control (IC) team is responsible for updating and maintaining vaccination records, while HR takes charge of documenting the health records of new employees and monitoring staff health/vaccination in compliance with MOH requirements. **Result:** The implementation of the Staff Health System offers several benefits as: (1) Empowerment of staff: The system grants employees the autonomy to schedule and reschedule vaccination and mask fitting sessions at their convenience; (2) Enhanced accessibility: Staff and other stakeholders can readily access vaccination and mask fitting records, as well as pre-employment lab results through the system; (3)Real-time data: The system can generate up-to-date data that are relevant to stakeholders and management, improving decision-making processes; (4) Resource efficiency: The digitalized system reduces the consumption of resources such as paper, toner, and manpower required for collating, updating, storing, and retrieving data; (5) Data accuracy: With the Staff Health System, data accuracy is ensured, reducing the risk of errors and discrepancies in records. **Conclusion:** Overall, the implementation of this digitalized solution has elevated Mt Alvernia Hospital’s service standards by enhancing efficiency and compliance in record-keeping and updating processes.

